# Jaundice without Bile Pigments in the Urine

**Published:** 1909-04-03

**Authors:** 


### JAUNDICE WITHOUT BILE PIGMENTS IN THE URINE.

A peculiar form of jaundice, characterised by yellow colouration of the integuments without any obvious elimination of bile pigments in the urine, notwithstanding the presence in the blood of pigment that gives Gmelin's reaction, was first described by Hayem in 1897. If it were not that the blood gives the nitric-acid test for bile pigments it might be thought that the yellow colour of these patients is due to something different to bile pigments—in short, that there is only apparent and not real jaundice. Be this as it may, the fact remains that patients come under observation from time to time, apparently mildly jaundiced in the ordinary way, and yet without demonstrable bile pigments in the urine.

The general characters of all Hayem's cases have been very similar. It has been a question of subicterus rather than of deep jaundice. The colouration of the integuments is usually a little different to the ordinary yellow, recalling rather that which has sometimes been termed xanthochromasia. In at least one case, however, the skin colour was indistinguishable from that of mild jaundice, and there was no particular restriction of the colour as regards its distribution. The urine not only does not give the ordinary tests for bile pigments, but it may actually be precisely the colour of healthy urine. In one of Hayem's cases there was a little urobilin, but this is found in other conditions besides jaundice.

The yellow colouration develops slowly and insidiously. Once established it persists, though it may appear to wax and wane. It is a chronic persistent "jaundice," mild in degree, sometimes more marked, sometimes less, yet always without bile pigments in the urine. Sometimes the liver is just palpable, smooth even, not hard and not painful nor tender to pressure. There is usually nothing to indicate gall-bladder trouble. The spleen is not enlarged. The fæces retain their normal colour.

Men seem to be affected more commonly than women. Whether alcoholism has anything to do with it is difficult to say. Most of the patients are dyspeptic, and they often present symptoms of actual gastritis. They are also apt to be of nervous temperament, with predominance of neurasthenic phenomena such as inaptitude at work, ready fatigue, tendencies to gloominess, irritability, and loss of weight. In view of the dyspeptic symptoms, it seems not unlikely that the icteric tint is due to secondary infection of the biliary passages from duodenitis, which so often accompanies gastritis, and the pancreatic ducts may possibly be affected in the same way.

